# Statistical challenges in assessing potential efficacy of complex interventions in pilot or feasibility studies

**DOI:** 10.1186/1745-6215-16-S2-O90

**Published:** 2015-11-16

**Authors:** Duncan T Wilson, Rebecca E A Walwyn, Julia Brown, Amanda J Farrin, Sarah R Brown

**Affiliations:** 1 Leeds Institute of Clinical Trials Research, Leeds, UK

## 

Early phase trials of complex interventions currently focus on assessing the feasibility of a confirmatory RCT and on conducting pilot work. These trials are not designed to enable a formal assessment of potential efficacy. As a result, guidance recommends any statistical analysis of treatment effects conducted in these trials should be treated with extreme caution and not used in deciding if a confirmatory trial of the intervention is warranted. Phase II trial designs developed in the drug context offer a potential solution, providing methods for selecting sample size parameters for feasibility and pilot trials which will enable formal assessments of potential efficacy to be carried out. In this paper we will outline the challenges encountered in extending ideas developed in the phase II drug trial literature to the complex intervention setting. The prevalence of multiple endpoints and clustering of outcome data will be identified as important considerations, having implications for timely and robust determination of optimal sample size parameters. The potential for Bayesian methods to help to identify robust trial designs and optimal decision rules will also be explored.

